# Post-stroke mania

**DOI:** 10.1192/j.eurpsy.2022.742

**Published:** 2022-09-01

**Authors:** M. Ortega

**Affiliations:** Icahn school at mount sinai - elmhurst hospital center, Psychiatry, elmhurst, United States of America

**Keywords:** mania, late onset, post-stroke, Elderly

## Abstract

**Introduction:**

Approximately one-third of stroke survivors develop poststroke depression. Post-stroke mania is relatively rare, with a prevalence of less than 2%. One review of case reports of late-onset mania in 2015 demonstrated that 51% of the patients had established vascular risk factors. In 28% of cases, the treatment of underlying organic cause contributed to successful remission of the manic episode.

**Objectives:**

For this review, we aimed to compile published case reports from the past 20 years to review late-onset mania as one of the neuropsychiatric outcomes of stroke and its management.

**Methods:**

literature search on Pubmed, PsychInfo, and Embase utilizing keywords combinations: Bipolar, Manic, Mania, Secondary, Stroke, Poststroke, Post-stroke, Elderly, Old, Late onset, Late-onset, Lateonset, Hemisphere, Brain, Vascular, Infarction.

**Results:**

Among the 17 case reports, the age of onset of manic episode ranged from 47 to 86 with a mean of 67 years. Of the 17 cases, the right hemisphere was the most frequently affected (14/17, 82%), with cerebrovascular lesion involving the left hemisphere in 3 cases (17.6%).

**Conclusions:**

Clinicians should consider mania secondary to an organic cause in patients presenting with focal or soft neurological signs or symptoms, manic episode with atypical symptoms such as visual or olfactory hallucinations, altered mental status, disorientation, impairment in memory or cognition, unusual age of onset for bipolar disorder, or unusual illness course such as single episode of mania or poor response to psychopharmacologic treatment. Some reviews suggest combination of mood stabilizers and second-generation antipsychotics. Benzodiazepines recommended as an adjunctive drug for acute management such as agitation, aggressive behavior or disinhibition.

**Disclosure:**

No significant relationships.

